# Transcriptomic characterization of the immunogenetic repertoires of heteromyid rodents

**DOI:** 10.1186/1471-2164-15-929

**Published:** 2014-10-24

**Authors:** Nicholas J Marra, J Andrew DeWoody

**Affiliations:** Department of Population Medicine and Diagnostic Sciences, Cornell University, S3-111 Schurman Hall, Tower Road, Ithaca, NY 14853 USA; Department of Forestry and Natural Resources, Purdue University, West Lafayette, IN 47907 USA; Department of Biological Sciences, Purdue University, West Lafayette, IN 47907 USA

**Keywords:** Transcriptomics, Spleen, Latitudinal diversity gradient, Differential gene expression, Evolutionary genomics

## Abstract

**Background:**

When populations evolve under disparate environmental conditions, they experience different selective pressures that shape patterns of sequence evolution and gene expression. These may be manifested in genetic and phenotypic differences such as a diverse immunogenetic repertoire in species from tropical latitudes that have greater and/or different parasite burdens than more temperate species. To test this idea, we compared the transcriptomes of one tropical species (*Heteromys desmarestianus*) and two species from temperate latitudes (*Dipodomys spectabilis* and *Chaetodipus baileyi*) from the Heteromyidae. We did so in a search for positive selection on sequences and/or differential expression, while controlling for phylogenetic history in our choice of species.

**Results:**

We identified 127,812 contigs and annotated 34,878 of these, identifying immune genes associated with interleukins, cytokines, and the production of mast cells. We identified 632 genes that were upregulated in *H. desmarestianus* (8.7% of genes tested) and 492 (6.7%) that were downregulated. Gene ontology terms including “immune response” were associated with 31 (4.9%) of the 632 upregulated genes. We found preliminary evidence for positive selection on three genes (Palmitoyltransferase ZDHHC5 Ubiquitin-conjugating enzyme E2 N, Krueppel-like factor 10, and Spindle and kinetochore-associated protein 1) along the *H. desmarestianus* lineage.

**Conclusions:**

Overall our findings pinpoint genes in species from disparate environments that are on different evolutionary trajectories in terms of expression levels and/or nucleotide sequence. Our data indicate there are significant differences in the expression of genes among the spleen transcriptomes of these species and that a number of these differentially expressed genes do not show the same pattern of differential expression in another tissue type. This points to the possibility of expression differences between these species specific to the spleen transcriptome.

**Electronic supplementary material:**

The online version of this article (doi:10.1186/1471-2164-15-929) contains supplementary material, which is available to authorized users.

## Background

Variation in natural selection between habitats can lead to the evolution of phenotypic differences between related taxa. For example, multiple studies have shown that freshwater populations of threespine stickleback, *Gasterosteus aculeatus*, consistently exhibit loss of body plate armor that is found in marine populations of the species and that there is evidence of a common genetic basis for these alternate phenotypes [1, 2]. Studies of environmental contrasts can provide an extreme but useful framework for identifying the impact of disparate selective pressures on the genome. For instance, the latitudinal diversity gradient (LDG), which describes the increase in species richness that occurs from the poles to the tropics, may be due to unstable climates and abiotic factors that constrain evolution in temperate latitudes or to the increased biotic interactions that drive diversity in tropical locales [3, 4]. For example, host-parasite interactions may act as powerful selective agents in tropical locales where overall species diversity is high.

Parasites (and predators) are known to impose greater selective pressure on their hosts in tropical environments than in temperate environments [5, 6]. This could be due in part to year round conditions conducive to high host productivity which supports high parasite diversity and abundance [7]. If selection due to parasite load is greatest in the tropics, or varies relative to temperate areas, tropical and temperate hosts should differ with respect to measures such as host infection status, immune response, and parasite abundance.

Several empirical studies have evaluated parasite abundance in temperate and tropical birds and found evidence of increased immune response and/or parasite richness at lower, tropical latitudes [8–10] although this directionality is not universal [11, 12]. Similarly, greater parasite load in tropical populations of the lizard *Eulamprus quoyii* has been reported [13]. In primates, a literature survey of pathogens and host ranges found evidence of the LDG for protozoan parasites but not for viruses [14], and within humans there is evidence of the LDG for several human parasites [15]. Finally, species richness in African ticks is correlated with the LDG as measured by their avian and mammalian hosts [16].

Evidence for immunogenetic divergence along an LDG exists as well. For instance, a study of genome-wide genetic differentiation (measured by *F*_*st*_) between tropical and temperate populations of *Drosophila melanogaster* found multiple immunity related genes that were significantly differentiated between the populations [17]. The same study also found enrichment for innate immunity genes involved in the *Toll* signaling pathway in the *F*_ST_ outliers indicative of possible selection on those loci. Another example is the pattern of increased MHC diversity in Atlantic salmon populations at lower latitudes and the fact that bacterial diversity is greater in rivers at lower latitudes, suggesting a possible link between increased pathogen abundance and genetic variation [18].

Whereas those earlier studies sought to quantify proximate selective pressures, we aimed to determine if selective pressures shaped sequence evolution and/or gross changes in gene expression of immune genes in a tropical rodent compared to temperate relatives. We used as evolutionary models the forest spiny pocket mouse (*Heteromys desmarestianus*) and two temperate relatives, *Chaetodipus baileyi* (Bailey’s pocket mouse) and *Dipodomys spectabilis* (banner-tailed kangaroo rat).

**Figure 1:**
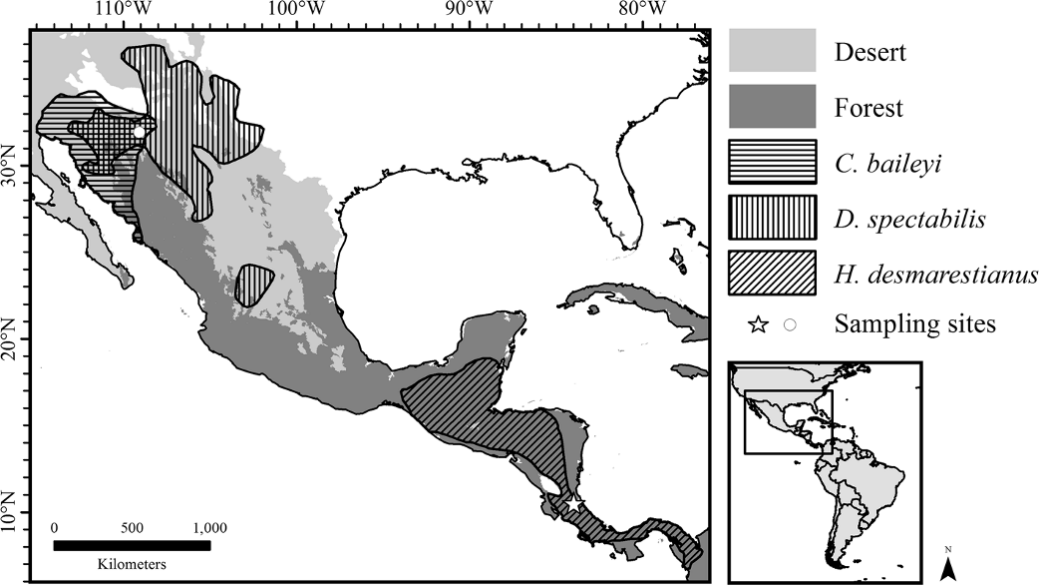
**Distribution and sampling locations of** ***Chaetodipus baileyi, Dipodomys spectabilis, and Heteromys desmarestianus.*** Range map of North America displaying habitat and latitude for all three study species constructed on Arc-GIS. Areas occupied by *C. baileyi* are highlighted by horizontal lines, areas occupied by *D. spectabilis* are denoted by vertical lines, and the range of *H. desmarestianus* is denoted by diagonal lines. Overlap between *C. baileyi* and *D. spectabilis* is cross hatched. The white star denotes the sampling location of *H. desmarestianus* at La Selva Biological Station, Costa Rica whereas the white circle marks the sampling location for *C. baileyi* and *D. spectabilis* at Portal, AZ, USA, Species range data were obtained from IUCN Red List of Threatened Species. Version 2013.1. [24]. Habitat data is from [25].

All three of our study species are members of the Heteromyidae, a family of new world rodents whose native range extends from North America through Central America and into northern South America [19, 20] (see Figure 1). This family is comprised of three subfamilies which diverged from one another roughly 22 mya [21]: the Heteromyinae*,* Perognathinae*,* and the Dipodomyinae (represented in our study by *H. desmarestianus*, *C. baileyi,* and *D. spectabilis*, respectively). *Heteromys* spp*.* have the most tropical latitudinal distribution of the family and are often found in lowland rainforest whereas *Chaetodipus* spp*.* and *Dipodomys* spp*.* generally have temperate desert distributions [19, 20, 22]. The historical biogeography of the species used in this study has not been described in detail, but Miocene fossils of the Perognathinae and Dipodomyinae ancestors have been found in the southwestern US and northern Mexico. These regions developed into arid deserts during the Pleistocene (historical biogeography of the Heteromyidae reviewed in [20]). No fossil record exists for *Heteromys* in Central America, but members of the genus are thought to have inhabited Central America prior to expanding into portions of northern South America as early as 3 mya [20, 23]. This history points to the long-term presence of these species in temperate and tropical latitudes, respectively.

Heteromyid rodents are afflicted by a variety of endo- and ectoparasites including viruses, bacteria, protozoa, nematodes, mites, ticks, lice, and other parasites [20]. Apparent parasite burdens increase as a species is more intensively studied [20] and this ascertainment bias is difficult to ameliorate. However, there are cases where our tropical study species (*H. desmarestianus*) appears to have a greater parasite diversity than its more temperate relatives. For example, sucking lice (*Fahrenholzia* species) are known to parasitize members of the Heteromyidae where a few individuals feed on the blood of a single host [26]. *H. desmarestianus* is one of the few host species in this complex that is parasitized by multiple sucking lice species [20, 26]. Overall, we expect that rodent species from more temperate latitudes have been infected with a different suite of parasites than the more tropical rodent species (i.e., *H. desmarestianus*).

To identify whether tropical species display molecular signatures of divergent selective pressure on immune response, we used a phylogenetic framework to compare these three rodent species. Our study framework provides a phylogenetic control due to our species selection. The Perognathinae and Heteromyinae are more closely related to each other than either are to the Dipodomyinae [19, 21]. Thus, we chose to compare a tropical species from the Heteromyinae to two species from temperate latitudes that were members of the Perognathinae and Dipodomyinae. These two species co-occur at our temperate latitude collection site and have similar ranges (Figure 1). By focusing on differences that were consistent between the tropical species and each of the temperate latitude species, any differences in gene expression and sequence evolution are more likely due to consistent life history differences between the two groups rather than phylogenetic divergence.

Our three study species have been the subject of a variety of ecological [27, 28], behavioral [29, 30], physiological [31–34], and phylogenetic studies [19, 21, 26]. Nevertheless, genomic resources for the group are limited and we have conducted transcriptome sequencing to expand the resources available to study various aspects of heteromyid biology. Previously, we sought to characterize the genetic basis of the superior osmoregulation and kidney function of *D. spectabilis* and *C. baileyi* [35]. There, we conducted RNA-seq of kidney tissue to identify consistent patterns of expression and selection in the kidney transcriptome of these two desert species relative to *H. desmarestianus* [35]. Here, we focus not on osmoregulation in the heteromyid kidney but on immunogenetic profiles of the heteromyid spleen. We do so in an effort to examine how selective pressures may drive transcriptomic differences between contrasting environments while simultaneously providing immunogenetic resources across the Heteromyidae.

This research forms a significant first step in identifying the presence and/or effects of a drastically different selective pressure on the tropical *H. desmarestianus* relative to the temperate *D. spectabilis* and *C. baileyi*, which are both constrained by adaptation to desert habitat [19, 35]. We used spleen tissue because its relative size has been used previously as a proxy for investment in immune defense [9] and because it has been used to characterize the major histocompatibility complex (MHC genes) in *D. spectabilis* [36]. The rodent spleen also functions in filtering and defense against blood borne pathogens (e.g. malarial parasites; reviewed in [37]) and the spleen has been implicated in both innate and adaptive immune response (reviewed in [38–40]). There is also evidence that gene expression in the spleen can be affected by the action of external pests/parasites feeding on hosts. For example, studies have shown that extracts from the salivary glands of sand and black flies can affect cell proliferation and gene expression in mouse spleen cells [41, 42]. Thus, we expect that gene expression in the spleen is sensitive to the variable immune challenges represented by different parasite assemblages on our species and that over time their spleen transcriptomes have been shaped by these different pressures.

## Methods

### Sample collection

The samples for this project were collected from the field as described in [43] and [35]. Briefly, spleen tissue from each of four adult *D. spectabilis* (2 males and 2 females) and four adult *C. baileyi* (2 males and 2 females) was harvested during collection trips to the same locale near Portal, AZ (about 31°37’N latitude) in December 2009 and December 2011, respectively. Additionally, spleen tissue was collected from four individual *H. desmarestianus* (1 male and 3 females) trapped within La Selva Biological Station in the lowlands of northern Costa Rica (roughly 10°25’N latitude) in June 2010. All 12 individuals were euthanized according to approved Purdue Animal Care and Use protocols, and tissue was harvested via dissections. Tissue was minced immediately and frozen in TRIzol^®^ reagent (Invitrogen) prior to transport back to Purdue University.

### RNA preparation and sequencing

At Purdue University, total RNA was extracted from a representative portion of each sample using TRIzol^®^ reagent (Invitrogen) according to manufacturer instructions. For the *D. spectabilis* and *H. desmarestianus* samples, ½ plate of 454 sequencing was conducted using cDNA synthesized from our initial RNA extractions with the ClonTech SMART cDNA synthesis kit [44] with a modified CDS III/3’ primer (see [43, 45, 46]). For Illumina sequencing, fresh RNA extractions were used to obtain total RNA for all 12 samples, which were then purified using RNA Clean and Concentrator columns (Zymo Research). RNA quality was evaluated with an Agilent 2100 Bioanalyzer before a uniquely barcoded 2 × 100 paired end library was constructed from each sample. All 12 libraries were then pooled and sequenced in two separate lanes of sequencing using an Illumina HiSeq 2000. This sequencing effort also included 12 libraries for a separate study [35], in total the same 24 libraries (12 of which were for this study) were sequenced in each lane of Illumina sequencing.

### Transcriptome assembly

Our assembly methodology (Table 1 lists programs and versions used here) closely follows our previous work on kidney transcriptomes [35]. Adaptors and library synthesis primers were trimmed from sequences and the libraries were then filtered to remove poor quality reads. DeconSeq version 0.4.1 [47] was used to remove reads with a minimum overlap of 60% and >80% similarity to the rRNA sequence. The filtered reads from all four individuals per species were pooled to assemble a species-specific *de novo* spleen reference transcriptome (n = 3 *de novo* assemblies) by first assembling all Illumina reads with the Trinity assembler [48]. Then Trinity contigs were fragmented into ≤1 kb pieces with a script from http://www.clarkfrancis.com/blast/fragblast.html before assembly with 454 reads in gsAssembler. The contigs from this gsAssembler assembly constituted our *de novo* transcriptome for each species.

**Table 1:** **List of programs used throughout the paper with a brief description of their function**

Program	Version	Use	Citation
DeconSeq	0.4.1	Read filtering and processing	[47]
Trinity	trinityrnaseq- r2013-02-25	*de novo* assembly of Illumina reads	[48]
gsAssembler	2.6	*de novo* assembly of Illumina and 454 reads	Roche/454 manual
Bowtie	1.0.0	Paired end read mapping implemented in RSEM	[49]
RSEM	1.2.0	Uses bowtie and maximum likelihood approaches to map reads to a scaffold and give the number of reads that map to a sequence	[50]
DESeq	1.14	Uses read counts to test for differential expression	[51]
MUSCLE	3.6	Multiple sequence alignment	[52]
PAML	4.7	Analysis of protein and DNA sequences using maximum likelihood based methods	[53, 54]

### BLAST annotation and GO enrichment

After filtering out all contigs that were ≤100 bp, we annotated the three resulting *de novo* transcriptomes with BLASTx [55] using the Swiss-Prot database and an e-value threshold of e ≤1 × 10^-6^ [46] to obtain annotated transcriptomes from the pool of four individuals per species. Using Blast2GO [56], we obtained gene ontology (GO) terms for our annotated contigs and conducted a Fisher’s exact test to identify gene ontologies that were consistently overrepresented or underrepresented in the *H. desmarestianus* dataset at p ≤ 0.05 relative to the other two species.

### Test for Differential Expression (DE)

As in [35], we obtained measures of gene expression for each of four individuals per species by mapping the individual Illumina reads back to our reference *de novo* transcriptomes that had been assembled in gsAssembler. Mapping was conducted with Bowtie [49] as run within the program RSEM [50]. We did so using the default parameters in the program RSEM [50]. RSEM allows a maximum of 2 mismatches per 25 bp seed but has Bowtie report all possible mapping locations for each read (reads that do not map are discarded). RSEM then uses this information in an algorithm to calculate the maximum likelihood estimate of the number of fragments assigned to each contig and thus account for reads that map to multiple possible locations [50, 57]. We rounded this value to the nearest whole number to assign a read count for individual contigs.

In principle, some genes could be covered by more than one contig due to several factors such as alternative splicing or incomplete coverage (gaps). Thus we summed read counts for all contigs with identical annotations to get a single read count for each gene. If ten or more reads from three of the four individuals mapped back to a gene then it was retained for DE analysis (otherwise it was discarded). Differential expression was evaluated between the three species by conducting the three possible pairwise analyses (*C. baileyi* vs. *H. desmarestianus*; *D. spectabilis* vs. *H. desmarestianus;* and *C. baileyi* vs. *D. spectabilis*) using the R package DESeq [51]. This program controls for library size, models the count data with a negative binomial distribution, and identifies significantly DE genes after a Benjamini-Hochberg correction for multiple testing and a threshold of an adjusted p (p_adj_) < 0.05 [51, 58]. Default parameters were used as described in the program’s documentation.

To gain greater insights into the gene transcripts, we tested for the enrichment of GO terms. First, we employed Fisher’s exact test to identify the GO terms that were enriched in genes overexpressed in *H. desmarestianus* relative to the two temperate species. Then, we performed another Fisher’s exact test to identify the GO terms that were enriched in genes underexpressed in *H. desmarestianus* relative to the two temperate species.

### Test for positive selection

Sequences were treated in a similar manner to [35] to build alignments of putative orthologues for four species (*M. musculus*, *C. baileyi*, *D. spectabilis*, and *H. desmarestianus*). After we obtained the best reciprocal BLAST hits for contigs from *D. spectabilis*, *C. baileyi*, and *H. desmarestianus,* we constructed sets of putative orthologues composed of one sequence from each species (three sequences per set of orthologues). We required all putative orthologues to have the same hit to known *Mus musculus* protein sequences (obtained from Ensembl release 75) during a BLASTx search with a cutoff of e-value <1 × 10^-9^. We further required that all species had a full-length alignment with the *M. musculus* sequence according to the BLASTx analysis. The coordinates of the full-length BLASTx search were used to trim each sequence of the orthologue set to the 5’ and 3’ positions of the alignment with *M. musculus*. We included the *M. musculus* coding sequence of the *M. musculus* protein hit as the fourth and final sequence for the set of orthologues.

All sets of four orthologous nucleotide sequences that remained after this filtering were aligned using MUSCLE v. 3.6 [52] in the first forward reading frame of the *M. musculus* coding sequence. These alignments were used in the codeml program in PAML 4.7 [53, 54] to test for selection along the tropical *H. desmarestianus* lineage. A guide tree was constructed from cytochrome *c* oxidase subunit I (*COI*) sequences from the four species; the three heteromyid *COI* sequences were from [21] (GenBank accession numbers: EF156845.1, EF156850.1, EF156856.1); *Mus musculus COI* sequence was from the *M. musculus* mitochondrial genome, GenBank accession: NC_005089.1). We allowed the branch lengths of the tree to be estimated by codeml for each alignment separately and we employed the clean data option to remove sites where gaps were encountered.

We used two tests to explicitly test whether the branch leading to the tropical species, *H. desmarestianus*, exhibited evidence of positive selection as indicated by an elevated ratio of nonsynonymous to synonymous changes (a dn/ds or ω >1). For each test we evaluated a model where ω was allowed to vary on a specified lineage vs. a null model where ω was the same for all lineages. First we conducted the ‘branch’ test [53, 54, 59, 60] where model 2 (the alternative model) is compared to model 0 (null model). In this case, two values of ω are estimated for the alternative model (one for a marked branch and one for the remaining lineages) and one value of ω is estimated for the entire tree in the null model. For our second comparison we allowed ω to vary across both lineages and sites. In this test we used model 2 as the alternative model with the additional modification of NSsites = 2 and compared this to a null model of model 2 and NSsites = 2 but where we fixed ω at a value of 1 for site class 2.

For a detailed explanation, see [61, 62] but briefly: under NSsites = 2 there are three possible classes for a site (site-classes 0, 1, and 2) and there are two branch types (background and foreground, the latter being our marked branch, *H. desmarestianus*). Each site class has its own value of ω that is estimated. In the null model, the value of site class 0 is 0 ≤ ω ≤ 1 for both branch types, the value of site class 1 is ω = 1 for both branch types, and the value of site class 2 is ω = 1 for the background branches and is fixed to be ω = 1 for the foreground branches as well. The alternative model is the same as the null model except that for site class 2 ω ≥ 1 is allowed for the foreground branch. This second comparison corresponds to the ‘branch-site’ models and we refer to it as the branch-sites test [61, 62].

These tests were conducted in a similar manner to those of [35] but here we tested for positive selection on the branch leading to the tropical species, *H. desmarestianus*. Positive selection was indicated by ω >1 and a significant difference between the tested and null models was indicated by a Likelihood Ratio Test (LRT). To assess significance we obtained a p-value for each LRT from the chi-squared distribution and then adjusted for multiple testing by applying a Benjamini-Hochberg correction for false discovery rate using the p.adjust function in R. Genes were identified as significant if they had an adjusted p-value (p_adj_) <0.05.

## Results

### Sequencing, assembly, and annotation

The sequences generated for this paper have been deposited in the NCBI-short read archive (NCBI SRA: Reads for NCBI BioProject: PRJNA261897, PRJNA261898, and PRJNA261902) and final transcriptome assemblies are available at Dryad http://dx.doi.org/10.5061/dryad.qn474. From the 2 lanes of Illumina sequencing we obtained 147,565,714 paired-end reads (2 × 100 bp reads) across all species. For the 454 pyrosequencing of *H. desmarestianus* and *D. spectabilis*, we obtained an additional 322,763 reads (mean length 294 bp; see Table 2). Mean contig length was roughly 1 kb for all three species (Table 3). With the GO term annotations from Blast2GO [56] we conducted a Fisher’s exact test to test for enrichment of GO terms in the *H. desmarestianus* dataset. There were 97 GO terms that were either enriched or underrepresented in the *H. desmarestianus* dataset, meaning there were either proportionally more or proportionally less genes (respectively) in the *H. desmarestianus* dataset with those GO terms than in the combined *D. spectabilis* and *C. baileyi* datasets. Of these, 73 GO terms were overrepresented and 24 were underrepresented in *H. desmarestianus* (see Additional file 1: Table S1). Several of these GO terms are closely linked to immunity including GO:0030368: interleukin-17 receptor activity; GO:0002215: defense response to nematode; GO:1900017: positive regulation of cytokine production involved in inflammatory response; and GO:0043306: positive regulation of mast cell degranulation. Although these terms are no longer significant after FDR correction, their raw p < 0.05 and more importantly their link to immunity indicate that the genes they describe may warrant further investigation.

**Table 2:** **Descriptive statistics from combined 454 and Illumina DNA sequencing runs of three heteroymid rodents**

	***Dipodomys spectabilis***	***Heteromys desmarestianus***	***Chaetodipus baileyi***
454 reads	203,096	119,667	N/A
mean 454 read length	292 bp	299 bp	N/A
Illumina reads	57,505,313	46,290,213	43,770,188

**Table 3:** **Summary of combined 454/Illumina assembly and annotation for contigs**

	***Dipodomys spectabilis***	***Heteromys desmarestianus***	***Chaetodipus baileyi***
# of contigs	58,435	35,318	34,059
Mean contig length	888 bp	1,158 bp	1,251 bp
Number of gene annotations	12,160	11,526	11,192
Annotated genes unique to the species	1,045	1,618	602

### Differential gene expression

**Figure 2:**
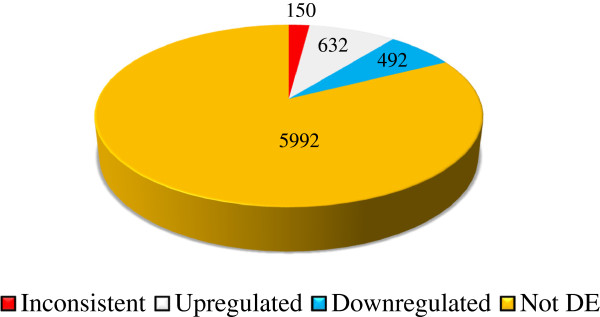
**Breakdown of genes tested for differential expression between tropical and temperate** ***heteromyid rodents*** **.** Tests for differential expression (DE) between the tropical species *Heteromys desmarestianus* and two related temperate species, *Dipodomys spectabilis* and *Chaetodipus baileyi.* 7,266 genes were tested for DE and 1,274 were significantly DE in both of these comparisons; significance was identified after a Benjamini-Hochberg correction.

From the DESeq analysis, out of 7,266 genes that passed the filtering step for the differential expression (DE) test, we identified 2,786, 2,533, and 2,893 significantly DE genes in the *D. spectabilis* vs. *H. desmarestianus*, *C. baileyi* vs. *H. desmarestianus*, and *C. baileyi* vs. *D. spectabilis* comparisons, respectively. The comparisons of evolutionary interest are the environmental contrasts whereby a temperate species (*C. baileyi* or *D. spectabilis*) is compared to the tropical species (*H. desmarestianus*). There were 1,274 genes that were DE in both of these comparisons. Of these, 49.6% (632 genes) were consistently upregulated (had higher expression) in *H. desmarestianus* relative to the temperate species and 38.6% (492 genes) were consistently downregulated, whereas 11.8% (150 genes) showed an inconsistent pattern of expression (upregulated in one comparison, downregulated in the other) (see Figure 2).

When applying a FDR correction (at a threshold of 0.05), the 632 genes that were consistently upregulated had a statistically significant overrepresentation of 78 GO terms and an underrepresentation of 48 GO terms. Table 4 contains the GO terms that were overrerpresented in the set of upregulated genes after filtering for the most specific GO level. This filtering removes higher order, less specific terms within a hierarchy of GO terms if all terms in the lineage of terms are enriched. For example, if a term such as ‘cell adhesion’ and the more specific term ‘positive regulation of cell adhesion’ were both enriched, then the filtering employed for Table 4 would remove the term ‘cell adhesion’ from the output. When evaluating the 492 genes that were consistently downregulated in *H. desmarestianus,* no terms were significantly overrerpresented or underrepresented.

**Table 4:** **GO terms significantly enriched in the set of genes that are upregulated in** ***Heteromys desmarestianus***

GO-ID	Term	Category	P-Value	BH FDR
GO:0030041	Actin filament polymerization	P	3.90E-04	4.58E-02
GO:0046165	Alcohol biosynthetic process	P	1.39E-04	2.20E-02
GO:0030154	Cell differentiation	P	3.66E-04	4.48E-02
GO:0034329	Cell junction assembly	P	3.29E-04	4.10E-02
GO:0032989	Cellular component morphogenesis	P	3.72E-04	4.51E-02
GO:0030198	Extracellular matrix organization	P	3.98E-04	4.58E-02
GO:0006775	Fat-soluble vitamin metabolic process	P	2.57E-04	3.32E-02
GO:0008610	Lipid biosynthetic process	P	4.11E-04	4.65E-02
GO:0045785	Positive regulation of cell adhesion	P	1.55E-04	2.30E-02
GO:0030335	Positive regulation of cell migration	P	1.04E-04	1.82E-02
GO:0014068	Positive regulation of phosphatidylinositol 3-kinase cascade	P	4.51E-05	9.49E-03
GO:0022603	Regulation of anatomical structure morphogenesis	P	2.20E-04	2.89E-02
GO:0060828	Regulation of canonical Wnt receptor signaling pathway	P	4.33E-04	4.82E-02
GO:0042306	Regulation of protein import into nucleus	P	4.58E-04	4.97E-02
GO:0035150	Regulation of tube size	P	1.79E-04	2.43E-02
GO:0009611	Response to wounding	P	1.00E-04	1.80E-02
GO:0048731	System development	P	5.66E-05	1.17E-02
GO:0003018	Vascular process in circulatory system	P	2.80E-05	6.85E-03
GO:0004872	Receptor activity	F	7.78E-06	2.65E-03
GO:0015629	Actin cytoskeleton	C	1.44E-06	6.80E-04
GO:0009925	Basal plasma membrane	C	1.29E-04	2.10E-02
GO:0009986	Cell surface	C	2.67E-07	1.74E-04
GO:0005911	cell-cell junction	C	1.54E-04	2.30E-02
GO:0030139	Endocytic vesicle	C	1.69E-04	2.37E-02
GO:0005615	Extracellular space	C	1.15E-04	1.95E-02
GO:0005887	Integral to plasma membrane	C	1.42E-04	2.21E-02
GO:0043005	Neuron projection	C	7.12E-05	1.39E-02

Terms listed here are filtered for specificity and all are significant after a Benjamini-Hochberg correction (BH FDR). P = biological process, C = cellular component, and F = molecular function.

### Selection tests

After filtering, there were 395 MUSCLE alignments of orthologous sequences as determined by our reciprocal BLAST analysis. The PAML analysis of this 395 gene dataset yielded 45 genes where model 2 varied from model 0 (p < 0.05, df = 1) for the branch test. Of these 45 genes, 4 were significant (p_adj_ < 0.05, df = 1) in the branch test after correction for multiple testing. This indicates that for these genes a model where the tropical lineage had a different ω from the rest of the tree was able to fit the data better than when one ω was applied to the whole tree. However, none of these 4 genes demonstrated an elevated ω >1 under model 2 and thus these 4 genes lacked evidence for positive selection.

The branch-sites test identified 8 genes with a substantial difference between the model which allowed selection and the null model (p < 0.05, df = 1). The test remained significant for one gene (Swiss-Prot symbol: *lrc8a*, gene symbol: *Lrrc8a*) after correction for multiple testing (p_adj_ < 0.05, df = 1), but this gene did not display an elevated ω indicative of positive selection. Of the other 7 genes that exhibited a preliminary difference in the branch-sites test, 6 had an ω >1 along the *H. desmarestianus* branch. We checked these 6 alignments by eye and discovered alignment errors in 3 of them. Thus, 3 genes remained that displayed an initial difference in the branch-sites test and exhibited elevated dn/ds ratios, indicating preliminary evidence for positive selection. However these 3 genes failed to remain significant following correction for multiple testing (Table 5).

**Table 5:** **Genes under positive selection according to the branch-site test**

Swiss-Prot symbol	Gene symbol	Protein name	dn/ds branch-site test	Number of sites	P-value branch-site test	P _adj_branch-site test
zdhc5	*Zdhhc5*	Palmitoyltransferase ZDHHC5 Ubiquitin-conjugating enzyme E2 N	66.65	2145	0.0159	1.00
klf10	*Klf10*	Krueppel-like factor 10	46.30	1434	0.0291	1.00
ska1	*Ska1*	Spindle and kinetochore-associated protein 1	65.38	759	0.0115	0.91

None were significant after FDR correction.

## Discussion

The role of selection in driving and shaping genetic variation has long been a subject of considerable interest in evolutionary biology. Some of the best molecular examples of natural selection come from the study of genes that play a role in immunity to infectious disease and/or pathogens [63, 64]. Indeed, a recent review found that 84 genes related to immunity exhibited interspecific signs of strong selection between the human and chimpanzee lineages [63].

The consistent signature of selection on genes associated with immunity, in conjunction with the latitudinal diversity gradient (LDG) and its logical corollary that increased biotic and host/parasite interactions are more prevalent in the tropics, led us to examine molecular differences between tropical and temperate rodents. For this we focused on developing immunogenetic resources for a group of non-model rodents that have representative species in both temperate and tropical latitudes. Previously, there have been cursory surveys of MHC diversity in the *D. spectabilis* genome [36]. However, genomic resources in this group are limited and those that do exist (e.g., a low-coverage genome in *D. ordii* [65]) are poorly annotated and too shallow for effective use in sequence assembly across the Heteromyidae [66]. Thus, we conducted RNA-sequencing to *de novo* assemble transcriptomes that we used to quantify gene expression and to test for divergent selection between the tropical and temperate species.

Our adult spleen transcriptome assemblies yielded a comparable number of contigs to our previous transcriptome work with these species [35] with slightly more in *D. spectabilis* (see Table 3). However, this increase in contig number is accompanied by a slightly shorter mean contig length due to a slightly more fragmented assembly. Regardless, we obtained an average of 24,594,286 reads per library (12,297,143 paired end reads per individual library) or 98,377,144 reads per species (49,188,572 paired end reads per species). BLAST annotation identified a mean of 11,626 genes per species, roughly 63% of which were expressed across all three species at levels high enough to pass our filtering for the DE test. Although not significant after FDR correction, the identification of several immunity related GO terms as overrepresented in the overall *H. desmarestianus* spleen transcriptome are noteworthy. In particular, the presence of GO terms such as GO:0002215: defense response to nematode are of interest considering that *Heteromys sp.* are known to be infected by nematodes and a new species of *Vexillata* was previously identified in *H. desmarestianus* [67].

Given the mean library size, we utilized DESeq [51] to test for DE because it is one of the more conservative methods for detecting DE [68] and is consistent with other approaches such as edgeR [35, 69]. 1,274 genes were significantly DE in each of two separate tests of the tropical *H. desmarestianus* versus *C. baileyi* and *D. spectabilis*. The DE genes showed a relatively consistent pattern with only 11.8% of genes switching direction of DE between the two comparisons (i.e., 88.2% did not change direction). We encountered 632 genes that were consistently upregulated in *H. desmarestianus* and 492 genes that were consistently downregulated in *H. desmarestianus*. The consistent DE pattern for these genes in the tropical *H. desmarestianus* relative to the temperate species is of great interest and may indicate expression patterns shaped by environmental factors.

Compared to our previous work with kidney transcriptomes of these species, fewer genes were DE between the spleen transcriptomes of the three species (1,274 here compared to 1,897 in kidney). Interestingly, there was relatively little overlap in the genes that were DE in *H. desmarestianus* spleen and the genes that were DE in our previous kidney transcriptome work (313 genes that were DE in both kidney and spleen). This leaves some 811 genes that had a consistent pattern of DE in spleen tissue between *H. desmarestianus* and each of the temperate latitude species that does not appear to be due to constitutive organismal differences in gene expression between the taxa. Clearly more tissues and individuals are needed to confirm this result, but patterns of DE in these 811 genes may be due to immunity related or spleen specific differences.

When considering the ontologies associated with the 632 genes upregulated genes, we found statistical overrepresentation or underrepresentation of 126 GO terms relative to the rest of the genes tested for DE. This contrasts with the absence of overrepresentation or underrepresentation of GO terms within the set of genes that were downregulated in *H. desmarestianus*. The 126 GO terms overrepresented or underrepresented in the upregulated genes include several implicated in the vertebrate immune response (e.g., response to wounding, endocytic vesicle, etc.). Further, when DE genes are queried specifically for GO terms such as “innate immune response” and “antigen presentation”, we find genes associated with innate and adaptive immune response, respectively. Specifically, 17 genes are annotated with “innate immune response” and 6 genes are annotated with “antigen presentation” in the set of 632 upregulated genes (see Additional file 1: Table S2 and S3). Overall, 31 of the 632 upregulated genes (4.9%) were annotated with one or more GO terms that contained the phrase “immune response”.

It may be that the predicted pathogen load encountered by tropical species has resulted in elevated expression of specific immunity related genes or DE of specific types of immune response genes (e.g. different emphasis on innate vs. adaptive immunity relative to temperate species). However, the DE patterns that we observed could result from other sources such as developmental differences (though we minimized this by sampling only adults for all species) or temporal patterns of expression. Clearly, more research is necessary to confirm their consistent upregulation in *H. desmarestianus* spleen across additional variables (e.g., time points, body conditions, parasite load, etc.).

Of additional interest is the consideration of these expression results in addition to the results of our selection tests. Our initial reciprocal best blast analysis yielded roughly 1,150 genes (both partial and full cds) that were putative orthologues across all three species. Following the *M. musculus* EST analysis, length filtering, and subsequent MUSCLE alignment, we obtained sequence alignments of 395 genes to test for evidence of positive selection. This represents roughly 3% of the genes that we annotated in the *H. desmarestianus* adult spleen transcriptome. Within these 395 genes, 35 (8.9% of genes tested for selection) were significantly DE, but none of these DE genes showed evidence of selection in our tests. We were able to detect departures from neutral models and detect cases where there was preliminary evidence of positive selection along the tropical *H. desmarestianus* branch (i.e., cases where the branch-sites test was significant before correction for multiple testing). We were mainly interested in those genes that not only showed departure from neutral models of sequence evolution, but those that also showed strong evidence of positive selection (elevated dn/ds) on the branch leading to *H. desmarestianus.* Such genes show divergence of the *H. desmarestianus* sequence from the other two species that served as temperate latitude environmental contrasts.

Automated sequence alignment can lead to an increased incidence of false positives [70, 71], so we required all of our putative orthologue sets to have full-length coverage of known *M. musculus* protein coding sequence. We also screened all alignments that indicated elevated dn/ds ratios along the *H. desmarestianus* branch and where the alternative model differed from the null initially (p < 0.05, df = 1). During this latter step, we ultimately removed three alignments that were confounded by gaps, resulting in three genes with preliminary evidence of selection before correction for multiple testing. Our strict and conservative filtering methods may have lowered detection of positive selection to a point where the occurrence of false positives is unlikely even though the existence of false negatives is probable. It is likely that the spleen transcriptome of *H. desmarestianus* contains additional genes that are under positive selection that were either removed from testing by our filtering, were not expressed at high enough levels to obtain full cds in all three species, could be heteromyid specific, or that could not be detected reliably using divergence based approaches. Ideally, further sequencing and another selection test with additional tropical and temperate heteromyids would be utilized to confirm the pattern observed herein and add statistical power to detect the extent of positive selection on the remaining 97% of the *H. desmarestianus* spleen transcriptome.

The three genes with some evidence of selection represent only 0.8% of our filtered set of genes used for the selection tests (395 genes), which falls well below other estimates of selection from genomic scans of divergence (e.g. 6% in [72], 8% in [73], and 20% in [74]). A consequence of our conservative filtering was that we were left with genes that were expressed at high enough levels to completely assemble full transcripts for all three species. This probable bias to highly expressed genes could have contributed to the lower incidence of positive selection in our data compared to the studies cited above that looked at a majority of the genes in the genome. It has been suggested that genes that are expressed across a wide range of tissues tend to have a lower rate of non-synonymous substitutions than genes with expression limited to one or a few tissues [75]. Indeed, when we compared the genes tested for selection here to those that were tested for selection in our previous study on the kidney transcriptomes of these species we found an overlap of 90 genes. The identification of these genes in additional tissues would be needed but these may be highly conserved genes that have expression across many organ systems and represent about 23% of the genes tested for selection herein.

A functional link between the gene and immunity is desirable in order to make the leap that these patterns of divergence between species are driven by parasite-(or pathogen) mediated selection in *H. desmarestianus*. There is no clear direct link between immune response and gene function for the genes listed in Table 5, and it is possible that the evolutionary pattern observed for these genes could be due to selective pressures other than immunity such as life history or habitat differences among these three heteromyids. For example, *H. desmarestianus* is native to wet areas, whereas the other two species inhabit xeric habitat [20, 35].

However, this lack of an obvious immune function does not preclude the possibility that selection is linked to immunity in some indirect way. Indeed, one textbook example of an immune related gene is selection on human β-globin. The top gene ontologies for this gene are heme binding, iron ion binding, oxygen binding, and oxygen transporter activity, and no immune function is apparent. However, we now know that the sickle cell allele for this gene is actively maintained in human populations due to the selective advantage of conferring malaria resistance [63, 76]. Further taxonomic sampling may confirm the selective significance of the genes listed in Table 5; these may indeed function in the immune response, but mechanistic studies are needed to evaluate this possibility. Of course, many of the other genes that are present in all three species (including those that were DE) may be under selection due to a function in immune response in the tropical species but require characterization of full-length coding sequences to be tested further.

## Conclusions

We have characterized the transcriptomes of three heteromyid rodents, identified a suite of genes that have been upregulated in *H. desmarestianus*, and identified several genes that exhibit preliminary evidence of positive selection. Their divergence from orthologues in temperate taxa could be driven by the selection pressure of elevated parasite abundance in tropical environments, be the result of other life history differences between these taxa, or be the result of stochastic processes. Further work is needed to combine direct estimates of sequence diversity for these and additional genes with estimates of parasite load and infection status in additional populations and species across a latitudinal gradient. The transcriptomes described herein would facilitate such an expanded study of sequence and expression differences between heteromyids from temperate and tropical latitudes. Additionally, these transcriptomes provide significant genetic resources from an immune tissue for members of all three of the heteromyid subfamilies.

### Availability of supporting data

The data sets supporting the results of this article are available in the NCBI Short Read Archive and DataDryad repository (details below):

- Illumina sequence reads: NCBI SRA: Reads can be found with the entries for NCBI BioProjects: PRJNA261897, PRJNA261898, and PRJNA261902.
- Final transcriptome assemblies, list of contigs, putative blast annotation, and R and perl scripts are available on Dryad, http://dx.doi.org/10.5061/dryad.qn474

## Authors’ information

NJM was formerly a graduate student and postdoctoral researcher in the Department of Forestry & Natural Resources at Purdue University. NJM is now a postdoctoral researcher in the Department of Population Medicine & Diagnostic Sciences at Cornell University. JAD was NJM’s advisor and is a Professor and University Faculty Scholar in the Department of Forestry & Natural Resources and the Department of Biological Sciences at Purdue University.

## Electronic supplementary material

Additional file 1: Table S1: List of GO terms from Fisher’s exact test for gene enrichment in the spleen transcriptome of *Heteromys desmarestianus* relative to *Dipodomys spectabilis* and *Chaetodipus baileyi*. Terms that are ‘over’ enriched are overrepresented in *H. desmarestianus*. FDR was > .05 for all terms so no terms were significant after Benjamini-Hochberg correction. **Table S2** List of genes that were upregulated in *Heteromys desmarestianus* and annotated with the GO term: “innate immune response”. **Table S3** List of genes that were upregulated in *Heteromys desmarestianus* and annotated with any GO term containing the phrase “antigen presentation”. (DOCX 22 KB)
