# Supplementary material for: Transcriptomic characterization of the immunogenetic repertoires of heteromyid rodents
Source: BMC Genomics. 2014 Oct 24;15(1):929. doi: 10.1186/1471-2164-15-929 (PMC4216838; doi:10.1186/1471-2164-15-929)
Supplement: Supplementary file 1 — Additional file 1: Table S1: List of GO terms from Fisher’s exact test for gene enrichment in the spleen transcriptome of Heteromys desmarestianus relative to Dipodomys spectabilis and Chaetodipus baileyi. Terms that are ‘over’ enriched are overrepresented in H. desmarestianus. FDR was > .05 for all terms so no terms were significant after Benjamini-Hochberg correction. Table S2 List of genes that were upregulated in Heteromys desmarestianus and annotated with the GO term: “innate immune response”. Table S3 List of genes that were upregulated in Heteromys desmarestianus and annotated with any GO term containing the phrase “antigen presentation”. (DOCX 22 KB) [file 12864_2014_6616_MOESM1_ESM.docx]

**Table S1**: List of GO terms from Fisher’s exact test for gene enrichment in the spleen transcriptome of *Heteromys desmarestianus* relative to *Dipodomys spectabilis* and *Chaetodipus baileyi*. Terms that are ‘over’ enriched are overrepresented in *H. desmarestianus*. FDR was >.05 for all terms so no terms were significant after Benjamini-Hochberg correction.

| **GO-ID** | **Term** | **Category** | **Raw P-Value** | **Enrichment** |
| --- | --- | --- | --- | --- |
| GO:0005833 | hemoglobin complex | Cellular Component | 1.81E-04 | over |
| GO:0033643 | host cell part | Cellular Component | 8.21E-03 | over |
| GO:0005858 | axonemal dynein complex | Cellular Component | 1.44E-02 | over |
| GO:0000015 | phosphopyruvate hydratase complex | Cellular Component | 3.20E-02 | over |
| GO:0042571 | immunoglobulin complex, circulating | Cellular Component | 3.23E-02 | over |
| GO:0005746 | mitochondrial respiratory chain | Cellular Component | 3.49E-02 | over |
| GO:0071546 | pi-body | Cellular Component | 3.56E-02 | over |
| GO:0032009 | early phagosome | Cellular Component | 3.96E-02 | over |
| GO:0005640 | nuclear outer membrane | Cellular Component | 4.22E-02 | over |
| GO:0005344 | oxygen transporter activity | Molecular Function | 7.26E-05 | over |
| GO:0031730 | CCR5 chemokine receptor binding | Molecular Function | 2.16E-02 | over |
| GO:0031683 | G-protein beta/gamma-subunit complex binding | Molecular Function | 2.89E-02 | over |
| GO:0008022 | protein C-terminus binding | Molecular Function | 3.00E-02 | over |
| GO:2001065 | mannan binding | Molecular Function | 3.13E-02 | over |
| GO:0004634 | phosphopyruvate hydratase activity | Molecular Function | 3.20E-02 | over |
| GO:0030368 | interleukin-17 receptor activity | Molecular Function | 3.23E-02 | over |
| GO:0008193 | tRNA guanylyltransferase activity | Molecular Function | 3.23E-02 | over |
| GO:0033130 | acetylcholine receptor binding | Molecular Function | 3.45E-02 | over |
| GO:0005388 | calcium-transporting ATPase activity | Molecular Function | 3.45E-02 | over |
| GO:0008137 | NADH dehydrogenase (ubiquinone) activity | Molecular Function | 3.47E-02 | over |
| GO:0015616 | DNA translocase activity | Molecular Function | 3.56E-02 | over |
| GO:0004648 | O-phospho-L-serine:2-oxoglutarate aminotransferase activity | Molecular Function | 3.56E-02 | over |
| GO:0004622 | lysophospholipase activity | Molecular Function | 4.17E-02 | over |
| GO:0097109 | neuroligin family protein binding | Molecular Function | 4.40E-02 | over |
| GO:0042799 | histone methyltransferase activity (H4-K20 specific) | Molecular Function | 4.47E-02 | over |
| GO:0048496 | maintenance of organ identity | Biological Process | 4.62E-04 | over |
| GO:0015671 | oxygen transport | Biological Process | 4.62E-04 | over |
| GO:0031448 | positive regulation of fast-twitch skeletal muscle fiber contraction | Biological Process | 1.79E-03 | over |
| GO:0097117 | guanylate kinase-associated protein clustering | Biological Process | 3.40E-03 | over |
| GO:2000311 | regulation of alpha-amino-3-hydroxy-5-methyl-4-isoxazole propionate selective glutamate receptor activity | Biological Process | 4.37E-03 | over |
| GO:0071908 | determination of intestine left/right asymmetry | Biological Process | 5.84E-03 | over |
| GO:0001812 | positive regulation of type I hypersensitivity | Biological Process | 8.85E-03 | over |
| GO:0006649 | phospholipid transfer to membrane | Biological Process | 1.28E-02 | over |
| GO:0097114 | N-methyl-D-aspartate receptor clustering | Biological Process | 1.32E-02 | over |
| GO:0097112 | gamma-aminobutyric acid receptor clustering | Biological Process | 1.42E-02 | over |
| GO:0002215 | defense response to nematode | Biological Process | 1.47E-02 | over |
| GO:0071909 | determination of stomach left/right asymmetry | Biological Process | 1.47E-02 | over |
| GO:0007158 | neuron cell-cell adhesion | Biological Process | 1.55E-02 | over |
| GO:0023041 | neuronal signal transduction | Biological Process | 1.68E-02 | over |
| GO:0045494 | photoreceptor cell maintenance | Biological Process | 1.82E-02 | over |
| GO:0033600 | negative regulation of mammary gland epithelial cell proliferation | Biological Process | 1.93E-02 | over |
| GO:0071625 | vocalization behavior | Biological Process | 1.93E-02 | over |
| GO:0045988 | negative regulation of striated muscle contraction | Biological Process | 2.00E-02 | over |
| GO:0042775 | mitochondrial ATP synthesis coupled electron transport | Biological Process | 2.08E-02 | over |
| GO:1900017 | positive regulation of cytokine production involved in inflammatory response | Biological Process | 2.29E-02 | over |
| GO:0035118 | embryonic pectoral fin morphogenesis | Biological Process | 2.57E-02 | over |
| GO:0071350 | cellular response to interleukin-15 | Biological Process | 2.96E-02 | over |
| GO:0001798 | positive regulation of type IIa hypersensitivity | Biological Process | 3.02E-02 | over |
| GO:0097459 | iron ion import into cell | Biological Process | 3.13E-02 | over |
| GO:2000821 | regulation of grooming behavior | Biological Process | 3.20E-02 | over |
| GO:0072189 | ureter development | Biological Process | 3.23E-02 | over |
| GO:0019369 | arachidonic acid metabolic process | Biological Process | 3.35E-02 | over |
| GO:0021860 | pyramidal neuron development | Biological Process | 3.45E-02 | over |
| GO:0061034 | olfactory bulb mitral cell layer development | Biological Process | 3.56E-02 | over |
| GO:0090076 | relaxation of skeletal muscle | Biological Process | 3.56E-02 | over |
| GO:0033601 | positive regulation of mammary gland epithelial cell proliferation | Biological Process | 3.57E-02 | over |
| GO:0032470 | elevation of endoplasmic reticulum calcium ion concentration | Biological Process | 3.69E-02 | over |
| GO:0033033 | negative regulation of myeloid cell apoptotic process | Biological Process | 3.77E-02 | over |
| GO:0061037 | negative regulation of cartilage development | Biological Process | 4.03E-02 | over |
| GO:0050919 | negative chemotaxis | Biological Process | 4.31E-02 | over |
| GO:0043306 | positive regulation of mast cell degranulation | Biological Process | 4.33E-02 | over |
| GO:0001662 | behavioral fear response | Biological Process | 4.39E-02 | over |
| GO:0060745 | mammary gland branching involved in pregnancy | Biological Process | 4.40E-02 | over |
| GO:1900139 | negative regulation of arachidonic acid secretion | Biological Process | 4.40E-02 | over |
| GO:1900138 | negative regulation of phospholipase A2 activity | Biological Process | 4.40E-02 | over |
| GO:0030178 | negative regulation of Wnt receptor signaling pathway | Biological Process | 4.49E-02 | over |
| GO:0051963 | regulation of synapse assembly | Biological Process | 4.52E-02 | over |
| GO:0043536 | positive regulation of blood vessel endothelial cell migration | Biological Process | 4.56E-02 | over |
| GO:0007186 | G-protein coupled receptor signaling pathway | Biological Process | 4.56E-02 | over |
| GO:0050912 | detection of chemical stimulus involved in sensory perception of taste | Biological Process | 4.68E-02 | over |
| GO:0090403 | oxidative stress-induced premature senescence | Biological Process | 4.69E-02 | over |
| GO:0090292 | nuclear matrix anchoring at nuclear membrane | Biological Process | 4.72E-02 | over |
| GO:0051247 | positive regulation of protein metabolic process | Biological Process | 4.84E-02 | over |
| GO:0032982 | myosin filament | Cellular Component | 2.75E-03 | under |
| GO:0016460 | myosin II complex | Cellular Component | 1.01E-02 | under |
| GO:0030934 | anchoring collagen | Cellular Component | 4.80E-02 | under |
| GO:0005958 | DNA-dependent protein kinase-DNA ligase 4 complex | Cellular Component | 4.80E-02 | under |
| GO:0004677 | DNA-dependent protein kinase activity | Molecular Function | 1.47E-02 | under |
| GO:0004190 | aspartic-type endopeptidase activity | Molecular Function | 1.73E-02 | under |
| GO:0004523 | ribonuclease H activity | Molecular Function | 4.02E-02 | under |
| GO:0052926 | dol-P-Man:Man(6)GlcNAc(2)-PP-Dol alpha-1,2-mannosyltransferase activity | Molecular Function | 4.83E-02 | under |
| GO:0052918 | dol-P-Man:Man(8)GlcNAc(2)-PP-Dol alpha-1,2-mannosyltransferase activity | Molecular Function | 4.83E-02 | under |
| GO:0016634 | oxidoreductase activity, acting on the CH-CH group of donors, oxygen as acceptor | Molecular Function | 4.88E-02 | under |
| GO:0051183 | vitamin transporter activity | Molecular Function | 4.88E-02 | under |
| GO:0043167 | ion binding | Molecular Function | 4.96E-02 | under |
| GO:0032933 | SREBP signaling pathway | Biological Process | 1.46E-02 | under |
| GO:0031642 | negative regulation of myelination | Biological Process | 1.67E-02 | under |
| GO:2000678 | negative regulation of transcription regulatory region DNA binding | Biological Process | 1.83E-02 | under |
| GO:0044342 | type B pancreatic cell proliferation | Biological Process | 2.12E-02 | under |
| GO:0048251 | elastic fiber assembly | Biological Process | 2.55E-02 | under |
| GO:0006308 | DNA catabolic process | Biological Process | 2.75E-02 | under |
| GO:0071214 | cellular response to abiotic stimulus | Biological Process | 2.92E-02 | under |
| GO:0035461 | vitamin transmembrane transport | Biological Process | 3.00E-02 | under |
| GO:0071616 | acyl-CoA biosynthetic process | Biological Process | 3.25E-02 | under |
| GO:0018200 | peptidyl-glutamic acid modification | Biological Process | 3.93E-02 | under |
| GO:0048853 | forebrain morphogenesis | Biological Process | 3.98E-02 | under |
| GO:0035337 | fatty-acyl-CoA metabolic process | Biological Process | 4.41E-02 | under |

**Table S2**: List of genes that were upregulated in *Heteromys desmarestianus* and annotated with the GO term: “innate immune response”

| Swiss-Prot symbol | Gene symbol | Protein name |
| --- | --- | --- |
| arpc5 | Arpc5 | actin-related protein 2 3 complex subunit 5 |
| birc1 | Naip | baculoviral iap repeat-containing protein 1 |
| casp1 | Casp1 | caspase-1 |
| cd180 | Cd180 | Lymphocyte antigen 78 |
| co2 | C2 | complement c2 |
| csf1r | Csfc1r | macrophage colony-stimulating factor 1 receptor |
| fgr | Fgr | tyrosine-protein kinase fgr |
| gab2 | Gab2 | grb2-associated-binding protein 2 |
| grap2 | Grap2 | grb2-related adapter protein 2 |
| irak3 | Irak3 | interleukin-1 receptor-associated kinase 3 |
| myo10 | Myo10 | unconventional myosin-x |
| nfac1 | Nfatc1 | nuclear factor of activated t- cytoplasmic 1 |
| pcbp2 | Pcbp2 | poly -binding protein 2 |
| pgfra | Pdgfra | platelet-derived growth factor receptor alpha |
| srpk2 | Srpk2 | srsf protein kinase 2 |
| tlr7 | Tlr7 | toll-like receptor 7 |
| xrcc6 | Xrcc6 | x-ray repair cross-complementing protein 6 |

**Table S3**: List of genes that were upregulated in *Heteromys desmarestianus* and annotated with any GO term containing the phrase “antigen presentation”

| Swiss-Prot symbol | Gene symbol | Protein name |
| --- | --- | --- |
| ap3b1 | Ap3B1 | ap-3 complex subunit beta-1 |
| dctn4 | Dctn4 | dynactin subunit 4 |
| fcgr1 | Fcgr1 | high affinity immunoglobulin gamma fc receptor i |
| itav | Itgav | integrin alpha-v |
| kif3c | Kif3c | kinesin-like protein kif3c |
| lyn | Lyn | tyrosine-protein kinase lyn |
